# Editorial: Hallmark of cancer: sustained proliferative signalling

**DOI:** 10.3389/fonc.2023.1328827

**Published:** 2023-11-23

**Authors:** Fabio Ciccarone, Maria Rosa Ciriolo

**Affiliations:** ^1^ Department of Biology, University of Rome ‘Tor Vergata’, Rome, Italy; ^2^ Laboratory of Biochemistry of Aging, IRCCS San Raffaele Roma, Rome, Italy

**Keywords:** cancer, metastasis, DNA damage response, metabolism, biomarkers

The first publications on the hallmarks of cancer date back two decades, and during the intervening years, the landscape of cancer research has changed significantly (1). Even though a set of criteria has been described for the characteristics that distinguish normal cells from cancer cells, they inevitably use similar metabolic networks. Oncogenic mutations and signaling pathways establish the correct orchestration of metabolic routes to allow cancer cells to replicate and survive in unsuitable tissue contexts, managing catabolic pathways for energy production and anabolism for biomass synthesis through changes in protein expression to enhance nutrient uptake, generate building block pathways, and release excretion products (2).

The first and most studied altered metabolism in cancer is aerobic glycolysis, often indicated by the Warburg effect, which refers to a high rate of glucose degradation irrespective of oxygen availability. Beyond aerobic glycolysis, other changes in metabolic pathways are also affected differently in subpopulations of cells of the same cancer and are also responsible for altered metabolic interactions with the microenvironment. An extensive reorganization of cell metabolism is, indeed, a prerequisite for neoplastic transformation and facilitates tumor progression and metastasis (3).

Upstream of metabolic changes, there are intricate growth signaling networks, the activation of which is sufficient to induce extensive changes in proliferative outcomes and metastatic potential. Cell signaling pathways are attractive for innovative chemotherapeutic tools, providing a plethora of different druggable targets that can exhibit long-reaching effects on cell survival, proliferation, and motility impinging on DNA damage response, metabolism, and tumor microenvironment remodeling (4). Thanks to the large number of high throughput studies available nowadays in different databases, including the Gene Expression Omnibus (GEO) and The Cancer Genome Atlas (TCGA), new informative gene signatures involved in cell signaling or any other molecular process can be characterized as diagnosis/prognosis type-specific or pan-cancer biomarkers (5).

Some of the abovementioned processes are covered in this Research Topic, which contains three Original Research articles and one Review.

The study by Aldaalis et al. demonstrated that the transcription factor sterol regulatory-element binding protein 1 (SREBP1) controls the cyclin D-CDK4/6-RB axis. SREBP1 binds to and activates the expression of cyclin D, thus inducing CDK4/6-mediated phosphorylation of RB and avoiding G1 cell cycle arrest. The upregulation of cyclin D was a signaling event downstream of SREBP1 activation due to low-cholesterol culture conditions and treatment with insulin. This work highlights how SREBP1, which is a well-known regulator of lipid biosynthesis in cancer cells, can manage the intimate connection between anabolism and proliferation, coupling the formation of new membranes necessary for cell division to cancer cell progression in the cell cycle.

Studies from Mao et al. and Wei et al. detailed the identification of new general or tumor-type specific biomarker genes/signatures. Mao et al. used public datasets to highlight the upregulation of matrix metalloproteinase-1 (MMP-1) expression in different types of cancer and its utilization as a prognostic biomarker based on patients’ overall survival and disease-free survival and as an immunological biomarker, assessing the correlation with several immune infiltration parameters. Experimental validation of the role of MMP-1 in the proliferation and survival of pancreatic cancer was included. Wei et al. focused on colorectal cancer (CRC) and on the DNA damage response (DDR) genes that are closely linked to its development/progression. A prognostic signature based on four DDR-related genes was developed and validated in different cohorts.

The review article by Werner and LeRoith focused on insulin-like growth factor 1 (IGF1), which represents one of the main signaling molecules involved in cell growth and development via activating the IGF1 receptor (IGF1R) signaling pathway. IGF1 is not regarded as a molecule able to induce mutations or transformation; however, it plays a fundamental role in enhancing the proliferation and survival of already transformed cells. Beyond its action in sustaining proliferative signaling, the activation of IGF1 signaling has been connected to most of the different hallmarks of cancer, with implications in genome instability, angiogenesis, and metastatization. Hints on the biological functions of nuclear IGF1R are also provided. The IGF1/IGFR1 pathway is a key example of how signaling events typically regulating normal cell activities can be exploited by cancerous cells to sustain their transformed/aggressive phenotype.

In conclusion, what emerges from this Research Topic is that, even though altered proliferation signaling is one of the first identified hallmarks of cancer, the intertwined pathways that control cell proliferation, metabolism, motility, and survival are far from being fully unraveled. The main issues concern the tissue-specificity of molecular pathways, cancer staging/grading, and the heterogeneity of the tumor microenvironment, including the clonal growth heterogeneity of cancer cells. Therefore, cell receptors and signaling proteins still represent appealing molecular targets to disable for cancer therapy in order to simultaneously affect processes sustaining tumor mass growth and its pro-tumoral microenvironment.

## Author contributions

MC: Conceptualization, Funding acquisition, Supervision, Validation, Writing – review & editing. FC: Conceptualization, Writing – original draft.
